# NAC domain transcription factors VNI2 and ATAF2 form protein complexes and regulate leaf senescence

**DOI:** 10.1002/pld3.529

**Published:** 2023-09-18

**Authors:** Isura Sumeda Priyadarshana Nagahage, Kohei Matsuda, Kyoko Miyashita, Sumire Fujiwara, Chanaka Mannapperuma, Takuya Yamada, Shingo Sakamoto, Toshiki Ishikawa, Minoru Nagano, Misato Ohtani, Ko Kato, Hirofumi Uchimiya, Nobutaka Mitsuda, Maki Kawai‐Yamada, Taku Demura, Masatoshi Yamaguchi

**Affiliations:** ^1^ Graduate School of Science and Engineering Saitama University Saitama Japan; ^2^ Department of Plant Physiology Umeå University Umeå Sweden; ^3^ Graduate School of Science and Technology Nara Institute of Science and Technology Ikoma Japan; ^4^ Bioproduction Research Institute National Institute of Advanced Industrial Science and Technology (AIST) Tsukuba Japan; ^5^ Umeå Plant Science Centre, Department of Plant Physiology Umeå University Umeå Sweden; ^6^ Global Zero‐Emission Research Center National Institute of Advanced Industrial Science and Technology (AIST) Tsukuba Japan; ^7^ Institute for Environmental Science and Technology Saitama University Saitama Japan; ^8^ Present address: College of Life Sciences Ritsumeikan University Kusatsu Japan; ^9^ Present address: Department of Integrated Biosciences, Graduate School of Frontier Sciences The University of Tokyo Kashiwa Japan

**Keywords:** *Arabidopsis thaliana*, leaf senescence, NAC domain protein, protein–protein interaction, transcription factor

## Abstract

The NAM, ATAF1/2, and CUC2 (NAC) domain transcription factor VND‐INTERACTING2 (VNI2) negatively regulates xylem vessel formation by interacting with another NAC domain transcription factor, VASCULAR‐RELATED NAC‐DOMAIN7 (VND7), a master regulator of xylem vessel formation. Here, we screened interacting proteins with VNI2 using yeast two‐hybrid assay and isolated two NAC domain transcription factors, *Arabidopsis thaliana* ACTIVATION FACTOR 2 (ATAF2) and NAC DOMAIN CONTAINING PROTEIN 102 (ANAC102). A transient gene expression assay showed that ATAF2 upregulates the expression of genes involved in leaf senescence, and VNI2 effectively inhibits the transcriptional activation activity of ATAF2. *vni2* mutants accelerate leaf senescence, whereas *ataf2* mutants delay leaf senescence. In addition, the accelerated leaf senescence phenotype of the *vni2* mutant is recovered by simultaneous mutation of *ATAF2*. Our findings strongly suggest that VNI2 interacts with and inhibits ATAF2, resulting in negatively regulating leaf senescence.

## 1 INTRODUCTION

NAC domain transcription factors, one of the plant‐specific transcription factor families, have been known to be associated with a wide variety of functions in plants, such as cell proliferation, biotic and abiotic responses, flowering time, secondary cell wall biosynthesis, and phloem development (Furuta et al., 2014; Kim et al., 2006; Podzimska‐Sroka et al., 2015; Souer et al., 1996; Yamaguchi et al., 2008). It has been reported that the function of several NAC domain transcription factors is post‐translationally regulated by modifications such as phosphorylation (Hamasaki et al., 2019), ubiquitination (Miao et al., 2016), and S‐nitrosylation (Kawabe et al., 2018; Ohtani et al., 2018). In addition, NAC domain transcription factors form homo‐dimer and/or hetero‐dimer complexes with other NAC domain transcription factors (Jensen & Skriver, 2014; Mohanta et al., 2020; Olsen et al., 2005; Puranik et al., 2012). In peaches, the NAC transcription factor BLOOD (BL) can interact with NAC1 to amplify its regulatory effect on anthocyanin biosynthesis (Zhou et al., 2015).

An Arabidopsis NAC domain transcription factor, VASCULAR‐RELATED NAC‐DOMAIN PROTEIN7 (VND7), acts as a master regulator of xylem vessel element differentiation (Nakano et al., 2015; Yamaguchi & Demura, 2010). Transcriptome analysis demonstrated that VND7 directly regulates a broad range of genes involved in xylem vessel differentiation (Yamaguchi et al., 2011). VND7 forms homo‐dimers and hetero‐dimers with other VND proteins (Yamaguchi et al., 2008). In addition, other NAC domain transcription factors, VND‐INTERACTING1 (VNI1), VNI2, and ANAC103, were isolated as interacting proteins with VND7 (Yamaguchi et al., 2015; Yamaguchi, Ohtani, et al., 2010). Further analyses showed that VNI2 inhibits the transcriptional activation activities of VND7 by forming protein complexes, negatively regulating xylem vessel formation (Ailizati et al., 2022; Yamaguchi, Ohtani, et al., 2010).

Promoter analysis demonstrated that *VNI2* expression was observed in various types of tissues and cells, such as phloem and guard cells as well as xylem vessel precursor cells (Yamaguchi, Ohtani, et al., 2010). In addition, *VNI2* expression was induced in response to abiotic stress or abscisic acid (ABA) treatment (Yang et al., 2011). These expression profiles suggested that VNI2 plays roles in various biological processes (Yamaguchi, Ohtani, et al., 2010; Yang et al., 2011). Indeed, a *vni2* mutant exhibited early leaf senescence and was susceptible to salt stress (Yang et al., 2011). It has also been reported that VNI2 interacts with a geminiviral replication initiator protein (Suyal et al., 2014). However, the molecular function of VNI2 remains to be fully elucidated.

Leaf senescence involves a series of intra‐cellular changes to breakdown chlorophyll and macromolecules in order to remobilize nutrients to reproductive, young, or even storage tissues. Some environmental changes such as drought, light, herbivores, or pathogens trigger leaf senescence (Sade et al., 2018). Regulation of leaf senescence involves a number of NAC domain transcription factors. Around 20 NAC domain transcription factors, including ORESARA1 (ORE1) (Kim et al., 2009), ORESARA1 SISTER1 (ORS1) (Balazadeh et al., 2011), AtNAP, ANAC046 (Oda‐Yamamizo et al., 2016), ATAF1 (Garapati et al., 2015), ANAC019 (Hickman et al., 2013), and ANAC072 (Li et al., 2016), are known to be positive regulators of leaf senescence. By contrast, JUNGBRUNNEN1 (JUB1) (Wu et al., 2012), ANAC017, ANAC082/VNI1, ANAC090 (Kim et al., 2018), and VNI2 (Yang et al., 2011) are reported as negative regulatory NAC domain transcription factors. These transcription factors consist of gene regulatory networks that control leaf senescence (Kim et al., 2014, 2018; Podzimska‐Sroka et al., 2015). Furthermore, it has been reported that these NAC domain transcription factors form various combinations of protein complexes during the leaf senescence process (Kim et al., 2018).

ATAF2 and ANAC102, which are close homologs of each other, belong to the stress‐responsive NAC subfamily SNAC‐A (Nakashima et al., 2012; Nuruzzaman et al., 2013). ANAC102 is involved in low‐oxygen stress responses (Christianson et al., 2009), high‐light stress responses (D'Alessandro et al., 2018), and brassinosteroid catabolism (Peng & Neff, 2021). It has also been reported that ANAC102 localizes in chloroplast nucleoids and regulates gene expression in chloroplasts (Xin et al., 2021). ATAF2 is involved in various processes such as biotic stress responses (Delessert et al., 2005), brassinosteroid catabolism (Peng et al., 2015; Peng & Neff, 2020, 2021), auxin biosynthesis (Huh et al., 2012), photomorphogenesis (Peng et al., 2015, 2020), circadian clock (Peng et al., 2015, 2020; Peng & Neff, 2020, 2021), and ethylene biosynthesis and responses (Peng et al., 2022). Recently, we found that ATAF2 upregulates several senescence regulators such as *ORE1*, *ORS1*, *VNI2*, and *ANAC046* (Nagahage et al., 2018, 2020). In addition, *ATAF2* overexpressing plants and *ataf2* mutants exhibited accelerated and delayed leaf senescence, respectively (Nagahage et al., 2020). Thus, these results demonstrated that ATAF2 promotes leaf senescence.

Here, to understand the biological roles of VNI2 more deeply, we screened for proteins that interact with VNI2. Then, we isolated two NAC domain transcription factors, ATAF2 and ANAC102. VNI2 interacts with and inhibits the transcriptional activation activities of ATAF2, as in the case of VND7. Furthermore, *ataf2* mutation suppressed the early leaf senescence phenotype observed in the *vni2* mutant. These data strongly suggested that VNI2 regulates leaf senescence through inhibition of ATAF2.

## 2 RESULTS

### 2.1 NAC domain proteins, ATAF2 and ANAC102, were isolated as interacting proteins with VNI2

**FIGURE 1:**
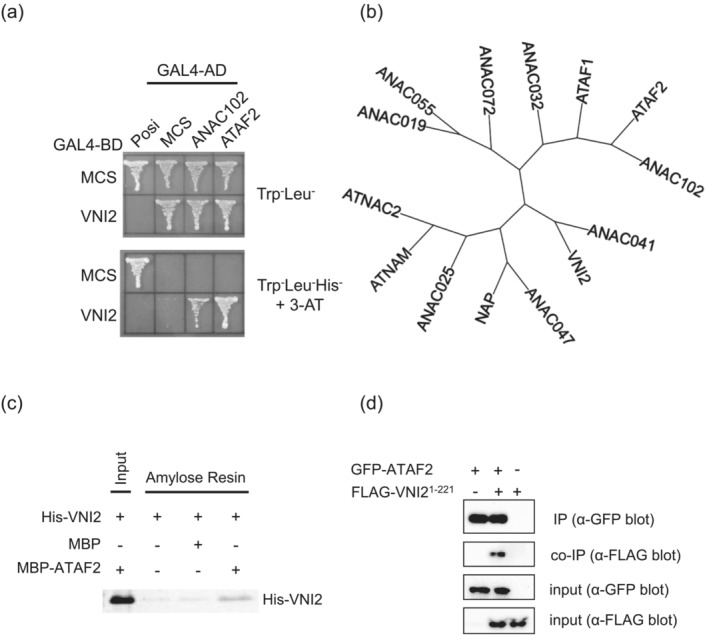
VNI2 interacts with ANAC102 and ATAF2. (a) Interaction of ANAC102 and ATAF2 together with VNI2 in yeast. The multi cloning site (MCS) and VNI2 fused to GAL4‐BD, and MCS,ANAC102, ATAF2 fused to GAL4‐AD were introduced into AH109 and grown on control (Trp‐Leu‐) and selective media (Trp‐Leu‐His‐). Plasmids containing MCS fused to GAL‐BD or GAL4‐AD were used as negative controls, and pBD‐wt and pAD‐wt were used as positive controls. (b) Phylogenetic tree of Arabidopsis NAC domain family involved in environmental stress. ANAC102 and ATAF2, isolated as interacting factors with VNI2, are closely related to each other. (c) In vitro binding of VNI2 to ATAF2. Proteins immobilized with the resin were subjected to immunoblot analysis. His‐VNI2 protein was detected with an anti‐His antibody. (d) In vivo binding of VNI2^1‐221^ and ATAF2. YFP‐ATAF2 and FLAG‐VNI2^1‐221^ were transiently co‐expressed in *Nicotiana benthamiana* leaves and subjected to co‐immunoprecipitation assay. Representative data from at least three replicates are shown.

An Arabidopsis NAC domain transcription factor, VNI2, negatively regulates xylem vessel formation by interacting with and inhibiting another NAC domain transcription factor, VND7, a master regulator of xylem vessel element differentiation (Yamaguchi, Ohtani, et al., 2010). Because *VNI2* expression was observed in various tissues and cells, and was regulated in response to endogenous and exogenous signals (Yamaguchi, Ohtani, et al., 2010; Yang et al., 2011), it is likely that VNI2 regulates some other biological processes besides xylem vessel formation. To further investigate the functions and biological roles of VNI2, we attempted to isolate proteins that interact with VNI2 using yeast two‐hybrid screening. Plant transcription factors often have transcription activities even in yeast cells (Yamaguchi et al., 2008). Thus, transcription factors lacking the transcriptional activation domain are usually used as bait for yeast two‐hybrid systems (Yamaguchi, Ohtani, et al., 2010). By contrast, budding yeast AH109 cells expressing full‐length VNI2 fused to the GAL4 DNA‐binding domain (GAL4‐BD‐VNI2) could not grow on selective medium lacking histidine with 1 mM 3‐AT (Figure 1a). Therefore, we used GAL4‐BD‐VNI2 as bait for screening. From at least 5 × 10^5^ independent cells transformed with the cDNA library prepared from Arabidopsis primary roots (Yamaguchi, Ohtani, et al., 2010), we identified four positive clones containing cDNAs encoding two distinct NAC domain transcription factors, ATAF2 and ANAC102 (3 clones). ATAF2 cDNA included the 5′ and 3′ untranslated regions and full‐length coding sequence, whereas all obtained ANAC102 cDNAs partly lacked the N‐terminal sequence but included the whole NAC domain (Figure S1). We confirmed that VNI2 also interacts with the full‐length coding region of ATAF2 or ANAC102 without untranslated regions (Figure 1a). ATAF2 and ANAC102 are the closest homologs in the Arabidopsis genome (Figure 1b). ATAF2 is known to be involved in biotic stress responses, brassinosteroid catabolism, auxin biosynthesis, light‐mediated development pathway together with the circadian clock, and leaf senescence (Delessert et al., 2005; Nagahage et al., 2018, 2020; Peng et al., 2015, 2020, 2022; Peng & Neff, 2020, 2021; Wang et al., 2009; Wang & Culver, 2012), whereas ANAC102 is involved in low‐oxygen responses and gene expression in chloroplasts (Christianson et al., 2009; Klok et al., 2002).

To investigate the transcriptional activities of ATAF2 and ANAC102, a transient expression assay was carried out (Figure S2). The effector constructs containing VND7, VNI2, ATAF2, or ANAC102 fused to GAL4‐BD under the control of *Cauliflower Mosaic Virus* (*CaMV*) *35S* promoter and reporter constructs containing firefly *Luciferase* (*LUC*) linked to GAL4‐binding sites were delivered to protoplasts obtained from Arabidopsis leaves. As previously reported, GAL4‐BD‐VND7 and GAL4‐BD‐ATAF2, but not GAL4‐BD‐VNI2, upregulated the LUC activity (Nagahage et al., 2018; Yamaguchi, Ohtani, et al., 2010). As with GAL4‐BD‐VNI2, GAL4‐BD‐ANAC102 did not upregulate the LUC activity (Figure S2).

### 2.2 ATAF2 interacts with VNI2 in vitro and in vivo

To understand which region of VNI2 is responsible for binding to ATAF2, yeast two‐hybrid screening was carried out using various lengths of VNI2 as bait (Figure S3). When GAL4‐BD fused VNI2^1‐170^, containing the whole NAC domain, was co‐transformed with GAL4‐AD‐ATAF2, the yeast cells grew on the selective medium. By contrast, transformants expressing GAL4‐BD‐VNI2^1‐138^ or GAL4‐BD‐VNI2^147‐252^, lacking parts of the NAC domain, together with GAL4‐AD‐ATAF2 were unable to grow on the selective medium (Figure S3). This result indicates that the whole NAC domain of VNI2 is required for binding to ATAF2.

To confirm the interaction observed in yeast cells, we conducted an in vitro pull‐down assay. ATAF2 fused to MBP‐ATAF2 and His‐VNI2 was prepared from *Escherichia coli* and incubated with amylose‐resin. Proteins bound to the amylose‐resin were subjected to immunoblot analysis with an anti‐His antibody. His‐VNI2 protein was effectively retained on the resin incubated with MBP‐ATAF2, whereas the resin immobilized with MBP hardly retained the His‐VNI2 (Figure 1c). These data indicated that ATAF2 binds to VNI2 in vitro.

To investigate the interaction in vivo, transient expression in *Nicotiana benthamiana* leaves was carried out. VNI2 has a PEST motif known to be the target of protein degradation. We previously demonstrated that VNI2 protein lacking the C‐terminal 30 amino acid region containing part of the PEST motif (VNI2^1‐221^) is more stable than the full length VNI2 (Yamaguchi, Ohtani, et al., 2010). When protein extracts from leaves expressing GFP‐fused ATAF2 (GFP‐ATAF2) and FLAG‐tagged VNI2^1‐221^ (FLAG‐VNI2^1‐221^) were immuno‐precipitated with anti‐GFP antibody, FLAG‐VNI2^1‐221^ was co‐precipitated (Figure 1d), indicating that VNI2 forms a protein complex with ATAF2 in plant cells.

### 2.3 VNI2 represses transcriptional activation activity of ATAF2

**FIGURE 2:**
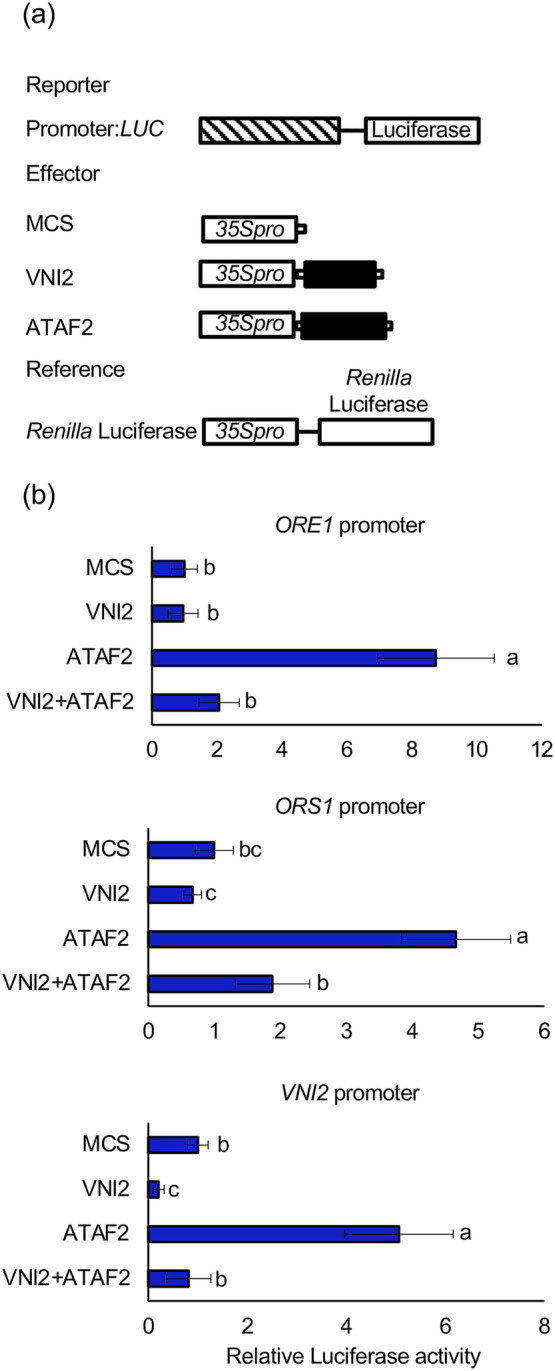
VNI2 inhibits the transcriptional activity of ATAF2. (a) Schematic diagram of the effector and reporter constructs. The reporter constructs contained the promoters of *ORE1*, *ORS1*, or *VNI2* and a firefly *LUCIFERASE* reporter gene. The effector constructs contained either the multi cloning site (MCS) fragment or *ATAF2* or full‐length *VNI2* downstream from the *CaMV35S* promoter. (b) Relative luciferase activities after transfection with individual reporter constructs and the effector constructs to protoplasts. Firefly luciferase activity was normalized using the activity of *Renilla* luciferase. Values and error bars indicate means ± SD (*n* = 4). Different letters indicate significant differences at *P* < .05, as determined by one‐way analysis of variance (ANOVA) with Tukey's post test.

ATAF2 regulates the expression of genes associated with leaf senescence, including *ORE1* and *ORS1* (Nagahage et al., 2020). In addition, it has been reported that VNI2 inhibits the transcriptional activation activity of VND7 by forming protein complexes (Yamaguchi, Ohtani, et al., 2010). To investigate how VNI2 regulates ATAF2 function, we performed a transient reporter gene expression assay using protoplasts of Arabidopsis mesophyll cells. Effector constructs containing *CaMV35S* linked to *VNI2* and *ATAF2* genes and reporter constructs containing firefly *LUC* driven by the *ORE1* or *ORS1* promoter were delivered into the protoplasts (Figure 2a). When ATAF2 was used as the effector, the reporter gene expression was increased compared to the control, as previously shown (Nagahage et al., 2020). Whereas, when both ATAF2 and VNI2 were used as the effectors, the reporter gene expression was comparable with the control (Figure 2b). These data suggested that VNI2 inhibits ATAF2 function by forming a protein complex, as in the case of VND7 (Yamaguchi, Ohtani, et al., 2010). It is noteworthy that ATAF2 upregulates *VNI2* expression (Nagahage et al., 2020). The transient assay showed that VNI2 also inhibited the reporter gene expression under control of its own promoter transactivated by ATAF2 (Figure 2b), suggesting the existence of negative feedback regulation for the *VNI2* gene. It is also noteworthy that when VNI2 was used as the effector, expression level of the *LUC* under controls of *VNI2* promoter was decreased compared to the control (Figure 2b). It might be possible that VNI2 interacts with and inhibits endogenous transcription factors.

### 2.4 Acceleration of age‐dependent leaf senescence in *vni2* is suppressed by *ataf2* mutation

**FIGURE 3:**
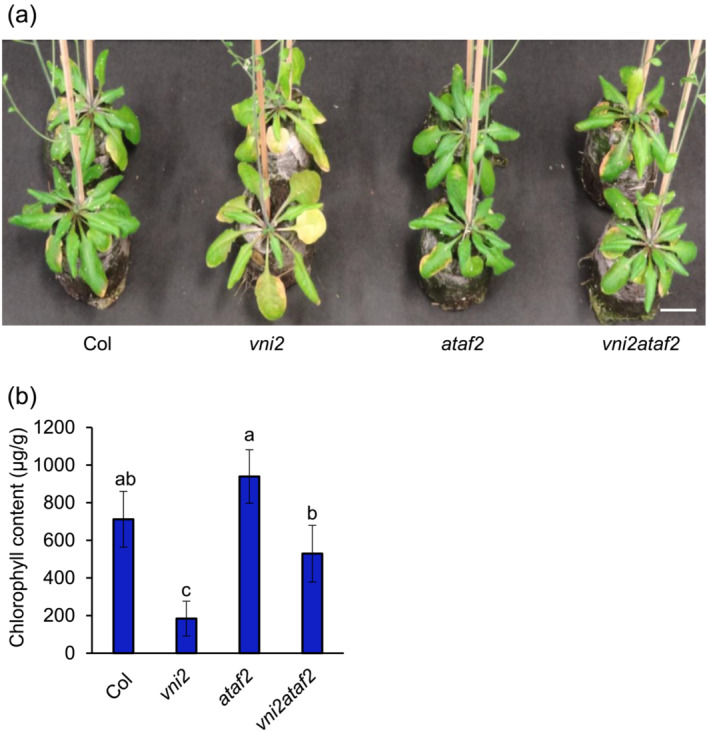
Effect of ATAF2 and VNI2 on age‐dependent leaf senescence. (a) 55‐day‐old wild‐type (Col), *vni2*, and *ataf2*, *vni2ataf2* plants grown under long‐day conditions. Bar = 2.5 cm. (b) Chlorophyll content of fifth leaves from 55‐day‐old plants. Error bars indicate SD (*n* = 5).

To examine genetic interactions between ATAF2 and VNI2, we observed loss‐of‐function mutants. RT‐PCR analysis showed that transcripts of *VNI2* or *ATAF2* were hardly detected in *vni2* and *ataf2* mutants, respectively (Figure S4). In addition, expression of both *VNI2* and *ATAF2* expression was not found in *vni2ataf2* double mutants (Figure S4). Under long‐day conditions, *vni2* mutants exhibited an accelerated leaf senescence phenotype compared with the wild‐type, as shown in a previous report (Yang et al., 2011) (Figure 3a). On the other hand, *ataf2* mutants did not show clear difference compared to the wild‐type. Interestingly, simultaneous mutations of *VNI2* and *ATAF2* attenuated the acceleration of leaf senescence as observed in *vni2* (Figure 3a). Consistent with the overall phenotypes, chlorophyll content was lowest in the *vni2* mutant, and that in *vni2ataf2* double mutants was comparable with that of the wild‐type (Figure 3b). These data indicated that decrease of the chlorophyll content in *vni2* mutants is dependent on the existence of ATAF2.

**FIGURE 4:**
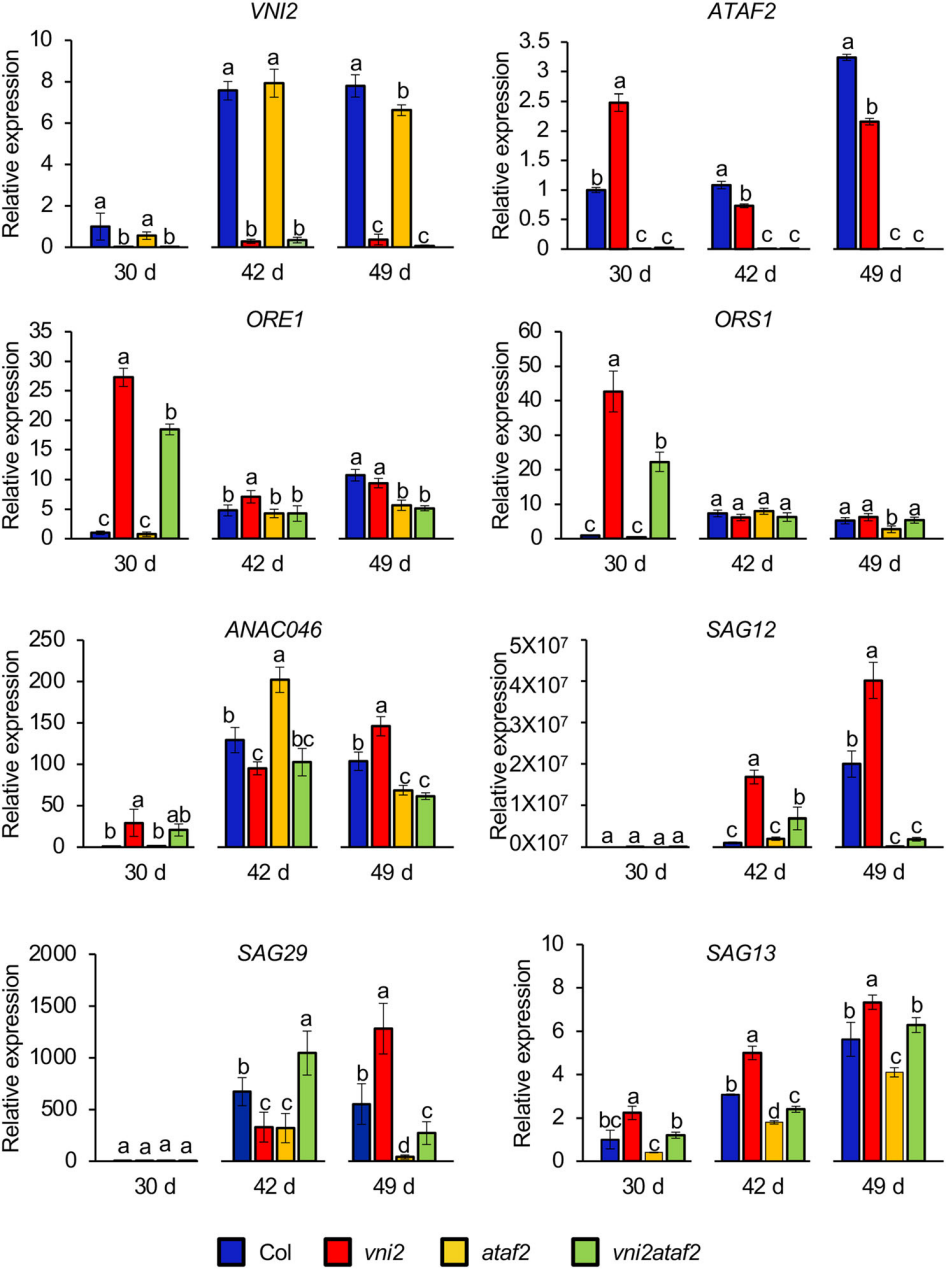
Expression analysis of the genes associated with leaf senescence. The fifth leaves of approximately 16 plants per each genotype were harvested at the indicated time points for total RNA extraction. Quantitative RT‐PCR analysis was performed, and four biological replicates were averaged. Values and error bars indicate means ± SD (*n* = 4). Different letters indicate significant differences at *P* < .05, as determined by one‐way analysis of variance (ANOVA) with Tukey's post test.

To investigate the expression levels of genes involved in leaf senescence, quantitative RT‐PCR was performed by using the fifth leaves of different ages of plants. In the wild‐type, increased expression of *VNI2* and *ATAF2* was observed in 42‐day‐old and 49‐day‐old plants, respectively, compared with 30‐day‐old plants (Figure 4). In addition, although *VNI2* expression was comparable between the wild‐type and *ataf2* mutant, the expression level of *ATAF2* in 30‐day‐old *vni2* plants was higher than that in wild‐type plants (Figure 4). Similarly, expression levels of *ORE1*, *ORS1*, and *ANAC046*, which are NAC domain transcription factors upregulated by ATAF2 (Nagahage et al., 2020), were higher in 30‐day‐old *vni2* mutants compared with the wild‐type, suggesting that VNI2 represses age‐dependent leaf senescence through inhibition of senescence‐associated genes regulated by ATAF2 (Figure 4). Interestingly, expression of *ORE1* and *ORS1* in 30‐day‐old *vni2ataf2* mutants was higher or lower than those of wild‐type plants or *vni2* mutants, respectively (Figure 4). Expression levels of *SAG12*, *SAG13*, and *SAG29/SWEET15* genes, known as senescence marker genes (Balazadeh et al., 2010; Matallana‐Ramirez et al., 2013), were lower in *vni2ataf2* mutants than those of *vni2* mutants at 49 days old (Figure 4). These results suggested that ATAF2 at least partly contributes to upregulation of the gene expression observed in *vni2* mutants. However, expression levels of *ANAC046* and *SAG29* in 42‐day‐old *vni2* mutants were lower than those of *vni2ataf2* mutants and/or wild‐type plants (Figure 4). It may be possible that *ANAC046* and *SAG29* genes are regulated by different transcription factors through the developmental stage.

### 2.5 Acceleration of dark‐induced leaf senescence in *vni2* is suppressed by *ataf2* mutation

**FIGURE 5:**
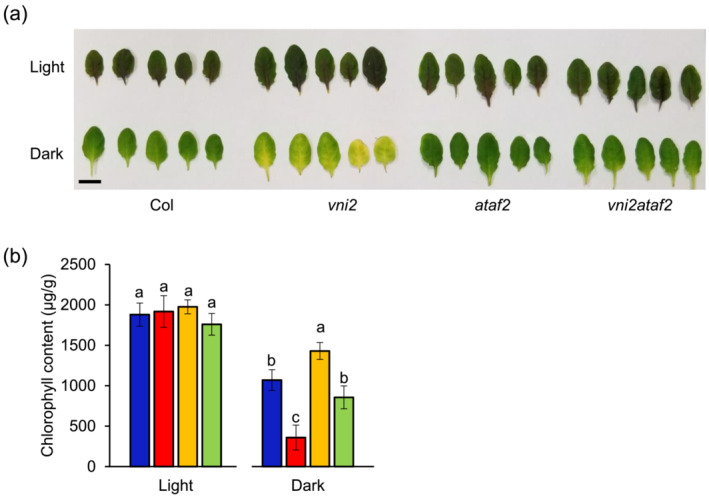
Dark‐induced leaf senescence in *vni2*, *ataf2*, and *vni2ataf2*. (a) The fifth leaves of 4‐week‐old plants incubated under continuous light or dark conditions for 5 days. Bar = 1 cm. (b) Chlorophyll content of the fifth leaves of 4‐week‐old plants incubated under continuous light or dark conditions for 5 days. Values and error bars indicate means ± SD (*n* = 5). Different letters indicate significant differences at *P* < .05, as determined by one‐way analysis of variance (ANOVA) with Tukey's post test.

ATAF2 promotes not only age‐dependent leaf senescence but also dark‐induced leaf senescence (Nagahage et al., 2020). Thus, we investigated the relationship of VNI2 and ATAF2 during dark‐induced leaf senescence. The fifth leaves from 4‐week‐old wild‐type, *vni2*, *ataf2*, and *vni2ataf2* mutants were incubated under continuous light or dark conditions for 5 days. No phenotypic changes were observed among the lines incubated under the light condition (Figure 5a). By contrast, under the dark condition, *vni2* mutants showed a leaf yellowing phenotype, and the leaves of *ataf2* mutants remained green. The leaves of *vni2ataf2* mutants were similar to those of wild‐type plants (Figure 5a). Consistent with the appearance, the lowest and highest chlorophyll contents were observed in *vni2* and *ataf2* mutants, respectively, and the chlorophyll contents of wild‐type and *vni2ataf2* double mutants were intermediate (Figure 5b).

**FIGURE 6:**
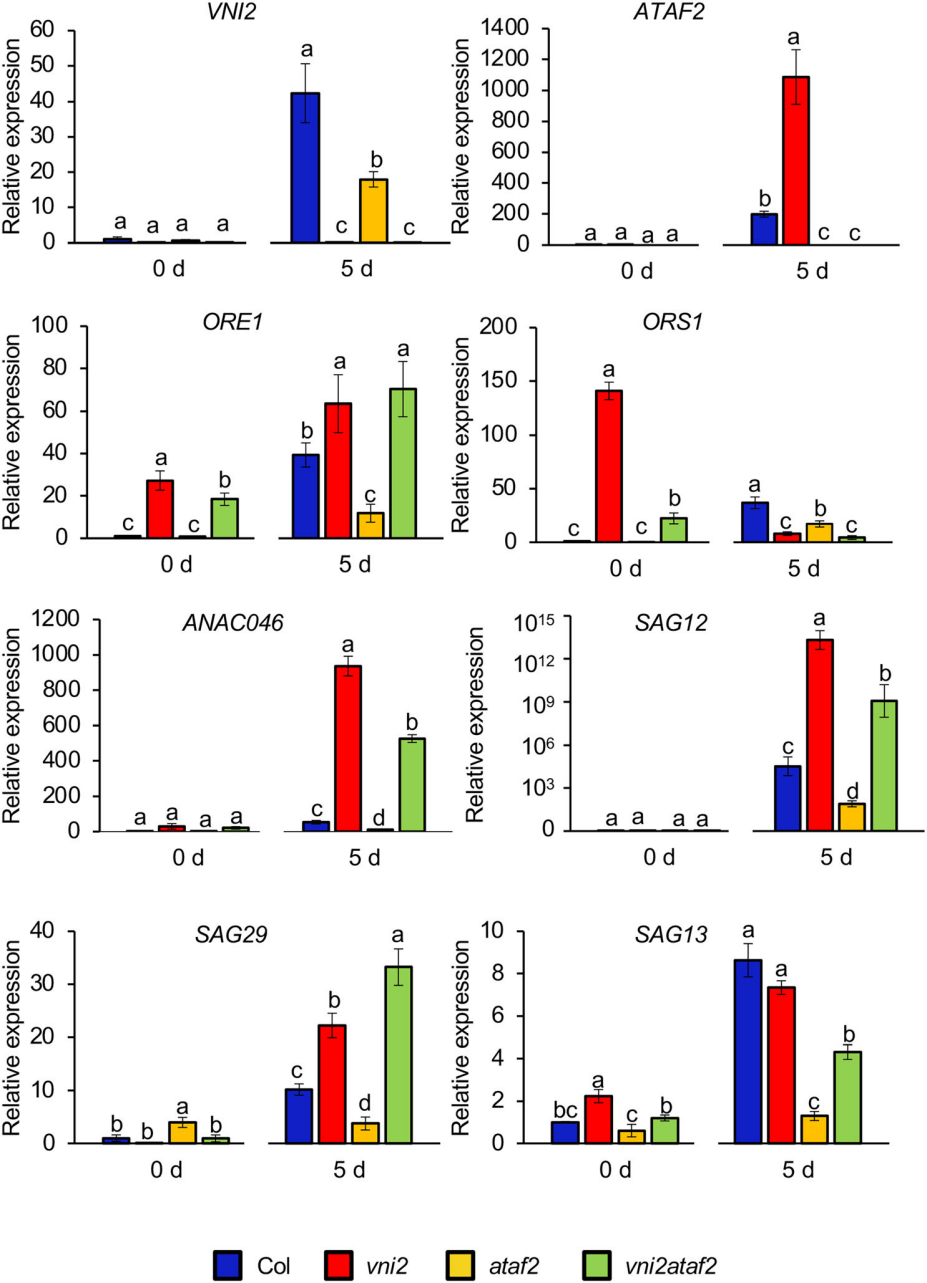
Expression of leaf senescence‐associated genes during dark‐induced senescence. The fifth leaves of approximately 16 plants per each genotype were harvested at the indicated time points for total RNA extraction. Quantitative RT‐PCR analysis was performed, and the biological replicates were averaged. Values and error bars indicate means ± SD (*n* = 4). Different letters indicate significant differences at *P* < .05, as determined by one‐way analysis of variance (ANOVA) with Tukey's post test.

To examine the expression levels of genes involved in leaf senescence under dark conditions, quantitative RT‐PCR was performed. In the wild‐type, expression levels of all tested genes were increased after dark incubation (Figure 6). In addition, after dark incubation, expression levels of *ORE1*, *ANAC046*, *SAG12*, and *SAG29* in *vni2* mutants and *ataf2* mutants were higher and lower, respectively, than those in the wild‐type (Figure 6). These data suggested that VNI2 represses the dark‐induced leaf senescence through inhibition of senescence‐associated genes regulated by ATAF2. Expression levels of *ORS1*, *ANAC046*, *SAG12*, and *SAG13* in *vni2 ataf2* mutants were lower than those in *vni2* mutants after dark incubation, consistent with the chlorophyll contents (Figure 6). By contrast, expression levels of *ORE1* and *SAG29* genes in *vni2ataf2* mutants were comparable with and higher than those of *vni2* mutants, respectively, after dark incubation (Figure 6). It is possible that VNI2 inhibits the transcriptional activities of some other NAC domain transcription factors that highly contribute to upregulation of *ORE1* and *SAG29* gene expression during dark‐induced leaf senescence.

## 3 DISCUSSION

### 3.1 VNI2 plays roles in leaf senescence and xylem vessel element differentiation by forming distinct NAC domain transcription factors

An NAC domain transcription factor, VNI2, was originally identified as an interacting factor with another NAC domain transcription factor, VND7, a key regulator of xylem vessel element differentiation (Yamaguchi, Ohtani, et al., 2010). VNI2 inhibits VND7 function, resulting in negative regulation of xylem vessel formation (Yamaguchi, Ohtani, et al., 2010). Here, we isolated other NAC domain transcription factors, ATAF2 and ANAC102, as interacting proteins with VNI2. ATAF2 and ANAC102 are known as stress regulatory NAC domain transcriptional factors and are closely related to each other. Yeast two‐hybrid analysis demonstrated that the whole NAC domain of VNI2 is necessary to interact with ATAF2 (Figure S3). Previously, it was reported that NAC subdomain V with the C‐terminal transcriptional activation domain of VNI2 was sufficient to interact with VND7 (Yamaguchi, Ohtani, et al., 2010), suggesting that VND7 and ATAF2 may bind to VNI2 in a different manner.

VND7 directly or indirectly regulates a broad range of genes involved in xylem vessel element differentiation (Yamaguchi et al., 2011; Zhong et al., 2010). Overexpression of *VND7* induces transdifferentiation into xylem vessel elements resulting from upregulation of the downstream genes (Kubo et al., 2005; Yamaguchi, Goue, et al., 2010). VNI2 inhibits the transcriptional activation activities of VND7 by forming protein complexes (Yamaguchi, Ohtani, et al., 2010). Previously, we showed that ATAF2 upregulates genes associated with leaf senescence (Nagahage et al., 2020). Overexpression of *ATAF2* promotes leaf senescence, whereas a knock‐out mutation of *ATAF2* exhibits delay of leaf senescence (Nagahage et al., 2020). As in the case of VND7, the transient gene expression assay demonstrated that VNI2 inhibits the transcriptional activation activities of ATAF2 (Figure 2). It is known that some transcription factors have several distinct roles by forming complexes with different proteins, such as DELLA family and PHYTOCHROME‐INTERACTING FACTOR (PIF) proteins (de Lucas et al., 2008; Feng et al., 2008; Yoshida et al., 2014). Likewise, our data suggested that VNI2 plays roles in different biological processes by binding to and inhibiting distinct NAC domain transcription factors. Xylem vessel element differentiation accompanies programmed cell death as well as secondary cell wall formation (Nakano et al., 2015; Yamaguchi & Demura, 2010). Leaf senescence is characterized by a reversible phase followed by an irreversible phase. Up to the irreversible phase, senescence is temporally inhibited by genetic programs competing with promoting factors for plants to adapt to the new environmental condition. However, beyond that point, leaf senescence cannot be reversed and progresses to leaf death (Guiboileau et al., 2010). It is possible that VNI2 has a common role to prevent progression to the irreversible stages during xylem vessel formation and leaf senescence by inhibiting the promoting factors.

### 3.2 Genetic relationship between VNI2 and ATAF2

Overexpression of VNI2 delays leaf senescence (Yang et al., 2011), whereas that of ATAF2 promotes leaf senescence (Nagahage et al., 2020). Conversely, single mutation of *VNI2* and *ATAF2* promotes and delays leaf senescence phenotypes, respectively (Nagahage et al., 2020; Yang et al., 2011). In this study, observation of single and double mutants suggested that the acceleration of the leaf senescence phenotype of *vni2* mutants is suppressed by *ataf2* mutation. It is likely that ATAF2 is released from inhibition of VNI2 in the *vni2* mutant background. However, *vni2ataf2* double mutants did not completely recover the acceleration of leaf senescence (Figures 3, 4, 5, 6). ATAF2 belongs to the stress‐responsive NAC subfamily SNAC‐A, which includes 6 other members (Nakashima et al., 2012; Nuruzzaman et al., 2013). It has been reported that members of the SNAC‐A subfamily also promote leaf senescence in response to environmental stresses (Garapati et al., 2015; Li et al., 2016; Mahmood et al., 2016; Takasaki et al., 2015). The phenotype of the *vni2ataf2* double mutant suggests a possibility that VNI2 also interacts with and inhibits some of the other SNAC‐A subfamily members. To prove the hypothesis, we should generate multiple mutants lacking *VNI2* and/or members of the SNAC‐A subfamily and observe the leaf senescence phenotypes of these mutants.

### 3.3 Transcriptional network during leaf senescence

**FIGURE 7:**
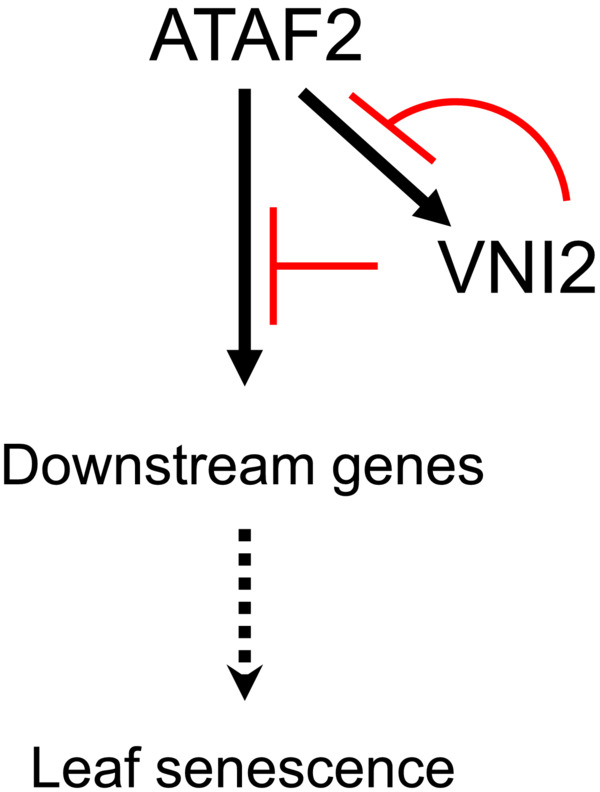
Schematic model of the relationship between ATAF2 and VNI2 during leaf senescence.

Recently, we have shown that VND7 negatively regulates *VNI2* expression (Ailizati et al., 2021). By contrast, ATAF2 upregulates *VNI2* gene expression (Nagahage et al., 2020). In this study, VNI2 inhibits its own expression that is activated by ATAF2 (Figure 2), indicating the negative feedback regulation for *VNI2* expression during the leaf senescence process (Figure 7). The INDETERMINATE DOMAIN (IDD) protein family activates the expression of the gibberellin‐positive regulator *SCARECROW‐LIKE 3* (*SCL3*), and SCL3 inhibits its own expression by forming protein complexes with IDD proteins (Yoshida et al., 2014). Likewise, some members of AUXIN RESPONSE FACTORs (ARFs) activate transcription of *Auxin* (*Aux*)/*INDOLE 3‐ACETIC ACID* (*IAA*) genes, while Aux/IAAs hetero‐dimerize with and inhibit ARFs (Freire‐Rios et al., 2020; Ito et al., 2016; Okushima et al., 2005). It is likely that the negative feedback regulations are important throughout plant development in response to various signals.

Leaf senescence is a well‐programmed process that includes macronutrient catabolism, detoxification, and transportation to young vegetative or reproductive parts and is essential for nutrient relocation and efficient plant growth. Thus, the senescence rate is an important aspect of the overall productivity of plants (Long et al., 2006). ATAF2 upregulates genes encoding not only promoting factors but also repressing factors of leaf senescence (Nagahage et al., 2020). It is possible that ATAF2 initiates leaf senescence by induction of promoting factors and maintains sufficient time for the nutrient recycling process by induction of *VNI2*. In addition, as described above, leaf senescence is an irreversible process. It is also possible that VNI2 induced by ATAF2 temporarily prolongs the reversible stages of leaf senescence. In order to confirm whether developmental or environmental conditions are sufficient to progress to the irreversible stage.

It has been reported that NAC domain transcription factors regulating leaf senescence form homodimer and/or heterodimer complexes (Kim et al., 2018). Taken together with these reports, our data also support the hypothesis that transcriptional regulations and various combinations of protein complexes among the NAC domain transcription factors play important roles in fine‐tuning the progression of leaf senescence.

In this study, we demonstrated that VNI2 interacts with and inhibits ATAF2, which is known to be involved in leaf senescence. We have shown that VNI2 regulates xylem vessel formation by forming the protein complex with VND7 (Yamaguchi, Ohtani, et al., 2010). Taken together with these reports, our data proposed a hypothesis that VNI2 plays roles in different biological events by forming protein complexes with distinct NAC domain transcription factors. As described above, regulation of leaf senescence involves a number of NAC domain transcription factors (Kim et al., 2014, 2018; Podzimska‐Sroka et al., 2015). Thus, it is necessary to investigate the relationship between VNI2 and these NAC domain transcription factors to understand the biological roles of VNI2 during leaf senescence more deeply. Furthermore, *VNI2* is expressed in various types of cells and stress conditions (Yamaguchi, Ohtani, et al., 2010; Yang et al., 2011). VNI2 may play roles in biological processes other than xylem vessel element differentiation and leaf senescence by forming protein complexes. A cDNA library composed only of cDNAs of Arabidopsis transcription factors has been established (Mitsuda et al., 2010). It is useful to identify novel interacting proteins with VNI2 to unveil other biological roles of VNI2.

## 4 EXPERIMENTAL PROCEDURES

### 4.1 Vector constructions

The promoter fragments and cDNAs were subcloned into the pENTR/D‐TOPO vector (Thermo Fisher Scientific) to generate entry clones. The resultant clones were integrated into GATEWAY destination vectors for yeast two‐hybrid assay (pBD‐GAL4‐GWRFC and pAD‐GAL4‐GWRFC; Yamaguchi et al., 2008), for expression of maltose‐binding protein (MBP) fusion proteins in *E. coli* (pMAL‐GWRFC; Yamaguchi, Ohtani, et al., 2010), for transient expression analysis (pAG35, pA35BDG, and pAGL; Endo et al., 2015; Yamaguchi et al., 2015), and for co‐immunoprecipitation (pGWB406 and pGWB412; Nakagawa et al., 2007). The GATEWAY destination vectors containing the nucleotide sequence of the multicloning site (MCS) fragments were used as controls (Yamaguchi et al., 2008). The nucleotide sequences of the MCS fragment and primers used in this study are shown in Table S1.

### 4.2 Plant materials

The Columbia ecotype of *Arabidopsis thaliana* (Col) was used for all the experiments. The *ATAF2* T‐DNA insertion mutant SALK_136355 and the *VNI2* T‐DNA insertion mutant SALK_143793 were obtained from the Arabidopsis Biological Resource Center. Plants were grown under long‐day conditions (16/8 h light/dark; 100 μmol m^−2^ s^−1^ light intensity) at 22°C. The *vni2ataf2* double mutant was generated by crossing *VNI2* and *ATAF2* T‐DNA lines.

### 4.3 Yeast two‐hybrid screening

The S.c. EasyCompTM Transformation Kit (Invitrogen) was used according to the protocol (https://www.lifetechnologies.com/order/catalog/product/K505001), and pBD‐GAL4‐GWRFC and/or pAD‐GAL4‐GWRFC plasmids were introduced into *Saccharomyces cerevisiae* strain AH109 by using S.c. solution III. Transformants were incubated at 30°C on MVD medium without tryptophan and leucine for 3 days. pBD‐wt and pAD‐wt harboring fragment C of lambda cI repressor (amino acid region 132–236) were used as positive controls (Agilent Technologies). Yeast successfully grown as above was transferred to MVD medium lacking tryptophan, leucine, and histidine and incubated at 30°C for 3 days. pBD‐GAL4‐MCS and pAD‐GAL4‐MCS were used as negative controls; 3AT (1 mM) was added to minimize the background false positive effect.

### 4.4 Phylogenetic tree analysis

An unrooted phylogenetic tree was constructed using the neighbor‐joining (NJ) method, and the bootstrap test was carried out with 1000 iterations for Arabidopsis NAC domain proteins.

### 4.5 Dual luciferase transient expression assay

Effector constructs, reporter constructs, and reference constructs were generated as described in Nagahage et al. (2020). Leaves of 3‐ to 4‐week‐old Arabidopsis plants without bolting were peeled using the Tape‐Arabidopsis Sandwich method (Sakamoto et al., 2016). The peeled leaves were subjected to an enzymatic solution (1% [w/v] Cellulase “Onozuka” R10 [Yakult Pharmaceutical Industry], .25% (w/v) macerozyme R10 [Yakult Pharmaceutical Industry], 400‐mM mannitol, 20‐mM MES [pH 5.7], 20‐mM KCl, 10‐mM CaCl_2_, and 5‐mM 2‐mercaptoethanol) for 1 h at 22°C with shaking at 60 rpm and then filtrated with 70‐μm nylon membran (Cellstraner 70 μm, BD Falcon). The protoplast cells were collected by centrifugation at 100 × ***g*** for 5 min and rinsed twice with W5 buffer (150‐mM NaCl, 125‐mM CaCl_2_, 5‐mM KCl, and 2‐mM MES [pH 5.7]) and incubated on ice for 10 min. The protoplast cells were resuspended with MMg buffer (400‐mM mannitol, 15‐mM MgCl_2_, and 4‐mM MES [pH 5.7]). Ten microliter plasmid solution was mixed with 35‐μl suspension of the protoplast cells and 45‐μl PEG solution (40% [w/v] PEG 4000 [Sigma‐Aldrich], 200‐mM mannitol, and 100‐mM CaCl_2_) and incubated for 10 min at room temperature. The protoplast cells were washed three times with W5 buffer and incubated for 16–20 h at 22°C under dark condition. Luciferase activity was measured with the Dual‐Luciferase Reporter Assay System (Promega, http://www.promega.com) using a Mithras LB940 Multimode Microplate Reader (Berthold, http://berthold.com) according to the manufactures' protocols.

### 4.6 In vitro pull‐down assay

Poly‐His tagged VNI2 (His‐VNI2) was prepared according to a previous report (Yamaguchi, Ohtani, et al., 2010). *ATAF2* cDNA was integrated into the pMAL‐GWRFC. MBP‐ATAF2 was expressed in *E. coli* strain BL21 *trxB* (DE3; Cosmo Bio) in the presence of isopropyl ß‐D‐thiogalactoside (IPTG) and purified with amylose resin (New England Biolabs). His‐VNI2 and/or MBP‐ATAF2 proteins were incubated with the amylose resin for 90 min at 4°C. The proteins immobilized with the resin were subjected to immunoblot analysis. His‐VNI2 protein was detected with an anti‐His antibody (Santa Cruz Biotechnology) and an anti‐rabbit IgG antibody (Amersham Biosciences).

### 4.7 In vivo co‐immunoprecipitation assay

Transient protein co‐expression in *N. benthamiana* and co‐immunoprecipitation assay were performed as described previously with some modifications (Fujiwara et al., 2014; Kim et al., 2007). Briefly, protein extracts were incubated with an anti‐GFP antibody (A‐11120, Thermo Fisher Scientific) and Dynabeads Protein A (Thermo Fisher Scientific) at 4°C for 1 h with gentle agitation. The proteins retained on the beads were separated by SDS‐PAGE. The blots were probed with the anti‐GFP antibody (ab6556, Abcam, 1:2000) and an anti‐DYKDDDDK tag antibody (019‐22394, Fujifilm, 1:20,000) and detected with ECL Prime (Cytiba Amersham).

### 4.8 Analysis of leaf senescence

The experiment was conducted as described previously in Nagahage et al. (2020). For dark‐induced leaf senescence analysis, the fifth rosette leaves of 4‐week‐old plants were excised and placed on moistened filter paper. The Petri dishes were sealed with surgical tape, wrapped with aluminum foil or not, and incubated at 22°C under continuous light conditions. Chlorophyll was extracted using dimethyl formamide at 4°C overnight. The chlorophyll extract was subjected to absorbance measurements via spectroscopy at wavelengths of 645 and 663 nm (Porra et al., 1989).

### 4.9 Gene expression analysis

For each genotype, the fifth leaves of about 16 plants were collected and averaged across four biological replicates. Total RNA was isolated by the RNeasy Plant Mini Kit (Qiagen) and treated with DNase I (Qiagen). The synthesis of cDNA was performed by using SuperScript II reverse transcriptase (Invitrogen). Quantitative RT‐PCR was performed by using Power SYBR Green PCR Master Mix and the 7300 Real‐Time PCR system (Applied Biosystems). The primers used for the expression analyses are listed in Table S1.

## AUTHOR CONTRIBUTIONS

Isura S.P. Nagahage and Masatoshi Yamaguchi conceived the original research plans. Isura S.P. Nagahage performed most of the experiments. Kohei Matsuda, Misato Ohtani, Ko Kato, Taku Demura, and Masatoshi Yamaguchi screened the interacting proteins with VNI2. Isura S.P. Nagahage conducted transient expression assays. Isura S.P. Nagahage and Takuya Yamada contributed to the pull‐down assay. Kyoko Miyashita and Sumire Fujiwara performed co‐immunoprecipitation assays. Chanaka Mannapperuma performed phylogenetic tree analysis. Isura S.P. Nagahage, Kyoko Miyashita, Sumire Fujiwara, Chanaka Mannapperuma, Takuya Yamada, Shingo Sakamoto, Toshiki Ishikawa, Minoru Nagano, Misato Ohtani, Ko Kato, Hirofumi Uchimiya, Nobutaka Mitsuda, Maki Kakawi‐Yamada, Taku Demura, and Masatoshi Yamaguchi interpreted the results. Isura S.P. Nagahage and Masatoshi Yamaguchi wrote the manuscript with input from all authors.

## CONFLICT OF INTEREST STATEMENT

The authors declare that they have no known competing interests that could have appeared to influence this work.

## Supporting information


**Data S1.** Peer Review.

**Figure S1.** Schematic diagram of NAC domain transcription factors. The gray boxes indicate subdomains I to V of the NAC domains. The black bar corresponds to the shortest encoded region of ANAC102 isolated by screening.

**Figure S2.** ATAF2 has transcriptional activation activity. (a) Schematic diagram of the constructs used in the dual luciferase transient assay. The reporter construct contained the firefly luciferase reporter gene under the control of five repeats of the upstream activation sequence of GAL4 (5 X GAL4 UAS) fused to a minimal *CaMV35S* promoter (min pro). The effector constructs contained GAL4‐BD bound to an empty multiple cloning site (GAL4‐BD‐MCS) or to coding sequences corresponding to full length *VND7*, *VNI2*, *ATAF2*, and *ANAC102* driven by the *CaMV35S* promoter (*35Spro*). (b) Results of the transient transfection assay. Firefly luciferase activity was normalized to *Renilla* luciferase activity. Error bars indicate SD (n = 4). Different letters indicate significant differences at *P* < .05, as determined by one‐way ANOVA with Tukey's post‐test.

**Figure S3.** The whole NAC domain of VNI2 is necessary for interaction with ATAF2. (a) Schematic diagram of full length and truncated VNI2 used for the yeast two‐hybrid assay. (b) Result of yeast two‐hybrid assay. Full length ATAF2 fused to GAL4‐BD, and full length or truncated VNI2 fused to GAL4‐AD were introduced into AH109 yeast cells. The transformed cells were grown on control (Trp^−^Leu^−^) and selective media (Trp^−^Leu^−^His^−^ with .1 mM 3‐AT). Plasmids containing MCS fused to GAL‐BD or GAL4‐AD were used as negative controls, and pBD‐wt and pAD‐wt were used as positive controls.

**Figure S4.** *VNI2* and *ATAF2* T‐DNA insertion lines. (a) Schematic diagram of the T‐DNA insertion sites of *vni2* and *ataf2*. Grey, black, and white boxes indicate untranslated regions, coding regions, and introns, respectively. Arrows indicate the locations of the primers used for RT‐PCR. (b) RT‐PCR analysis results. Analysis was performed using the seedlings of 7‐day‐old plants.

**Table S1.** Oligonucleotides used in this study.

## Data Availability

The data that support the findings of this study are available from the corresponding authors upon reasonable request.
