# Supplementary figures and images for: NAC domain transcription factors VNI2 and ATAF2 form protein complexes and regulate leaf senescence
Source: Plant Direct. 2023 Sep 18;7(9):e529. doi: 10.1002/pld3.529 (PMC10507225; doi:10.1002/pld3.529)

## Slide 1
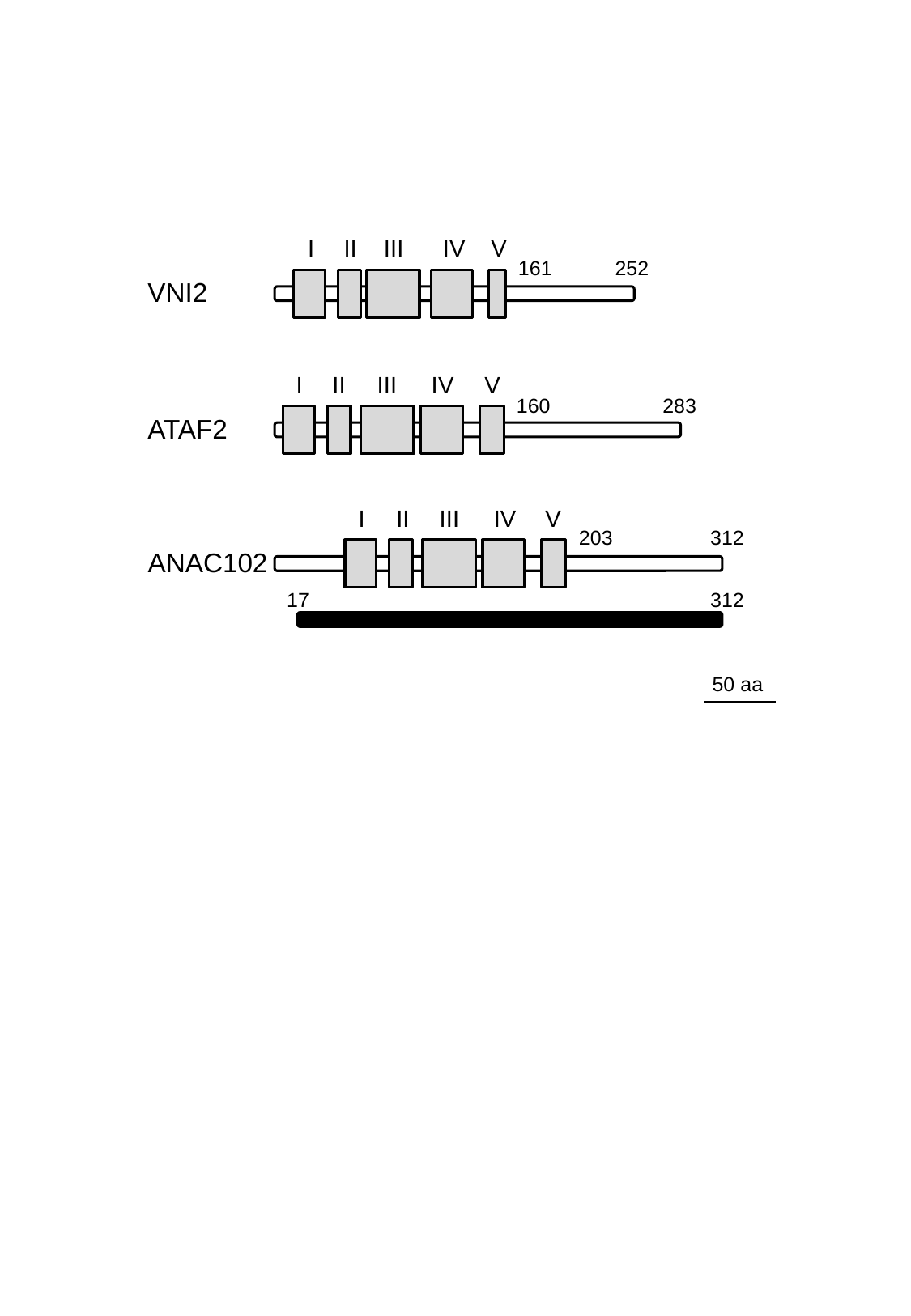

I
II
III
IV
V
161
252
VNI2
I
II
III
IV
V
160
283
ATAF2
I
II
III
IV
V
203
312
ANAC102
17
312
50 aa

Supplement: Supplementary file 2 — Figure S1. Schematic diagram of NAC domain transcription factors. The gray boxes indicate subdomains I to V of the NAC domains. The black bar corresponds to the shortest encoded region of ANAC102 isolated by screening. [file PLD3-7-e529-s001.pptx]

## Slide 1
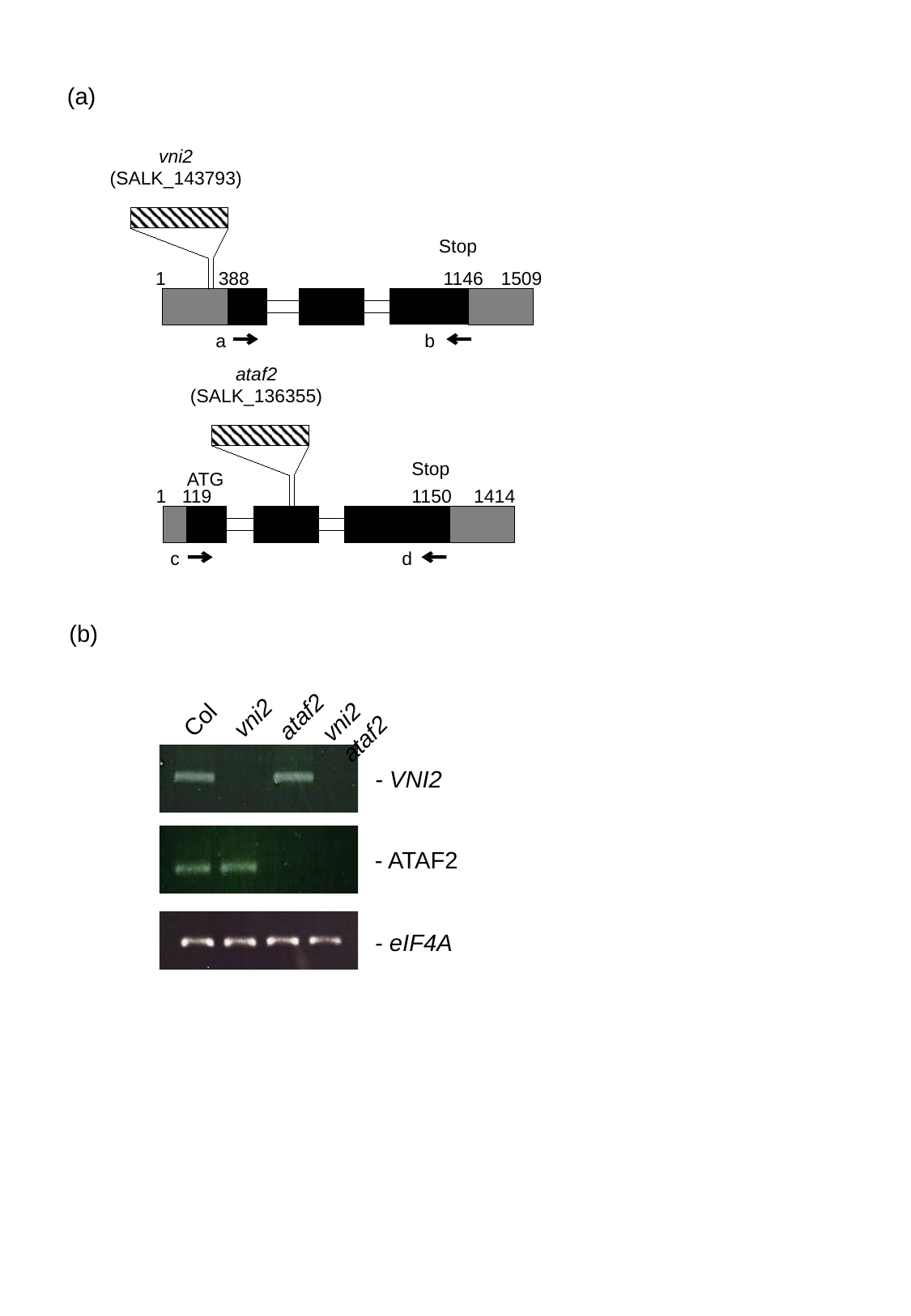

(a)
vni2
(SALK_143793)
Stop
1
388
11460
1509
a
b
ataf2
(SALK_136355)
Stop
ATG
1
119
1150
1414
c
d
(b)
vni2 ataf2
Col
vni2
ataf2
- VNI2
- ATAF2
- eIF4A

Supplement: Supplementary file 5 — Figure S4. VNI2 and ATAF2 T‐DNA insertion lines. (a) Schematic diagram of the T‐DNA insertion sites of vni2 and ataf2. Grey, black, and white boxes indicate untranslated regions, coding regions, and introns, respectively. Arrows indicate the locations of the primers used for RT‐PCR. (b) RT‐PCR analysis results. Analysis was performed using the seedlings of 7‐day‐old plants. [file PLD3-7-e529-s006.pptx]
