# Supplementary material for: NAC domain transcription factors VNI2 and ATAF2 form protein complexes and regulate leaf senescence
Source: Plant Direct. 2023 Sep 18;7(9):e529. doi: 10.1002/pld3.529 (PMC10507225; doi:10.1002/pld3.529)
Supplement: Supplementary file 3 — Figure S2. ATAF2 has transcriptional activation activity. (a) Schematic diagram of the constructs used in the dual luciferase transient assay. The reporter construct contained the firefly luciferase reporter gene under the control of five repeats of the upstream activation sequence of GAL4 (5 X GAL4 UAS) fused to a minimal CaMV35S promoter (min pro). The effector constructs contained GAL4‐BD bound to an empty multiple cloning site (GAL4‐BD‐MCS) or to coding sequences corresponding to full length VND7, VNI2, ATAF2, and ANAC102 driven by the CaMV35S promoter (35Spro). (b) Results of the transient transfection assay. Firefly luciferase activity was normalized to Renilla luciferase activity. Error bars indicate SD (n = 4). Different letters indicate significant differences at P < .05, as determined by one‐way ANOVA with Tukey's post‐test. [file PLD3-7-e529-s002.pptx]

## Slide 1
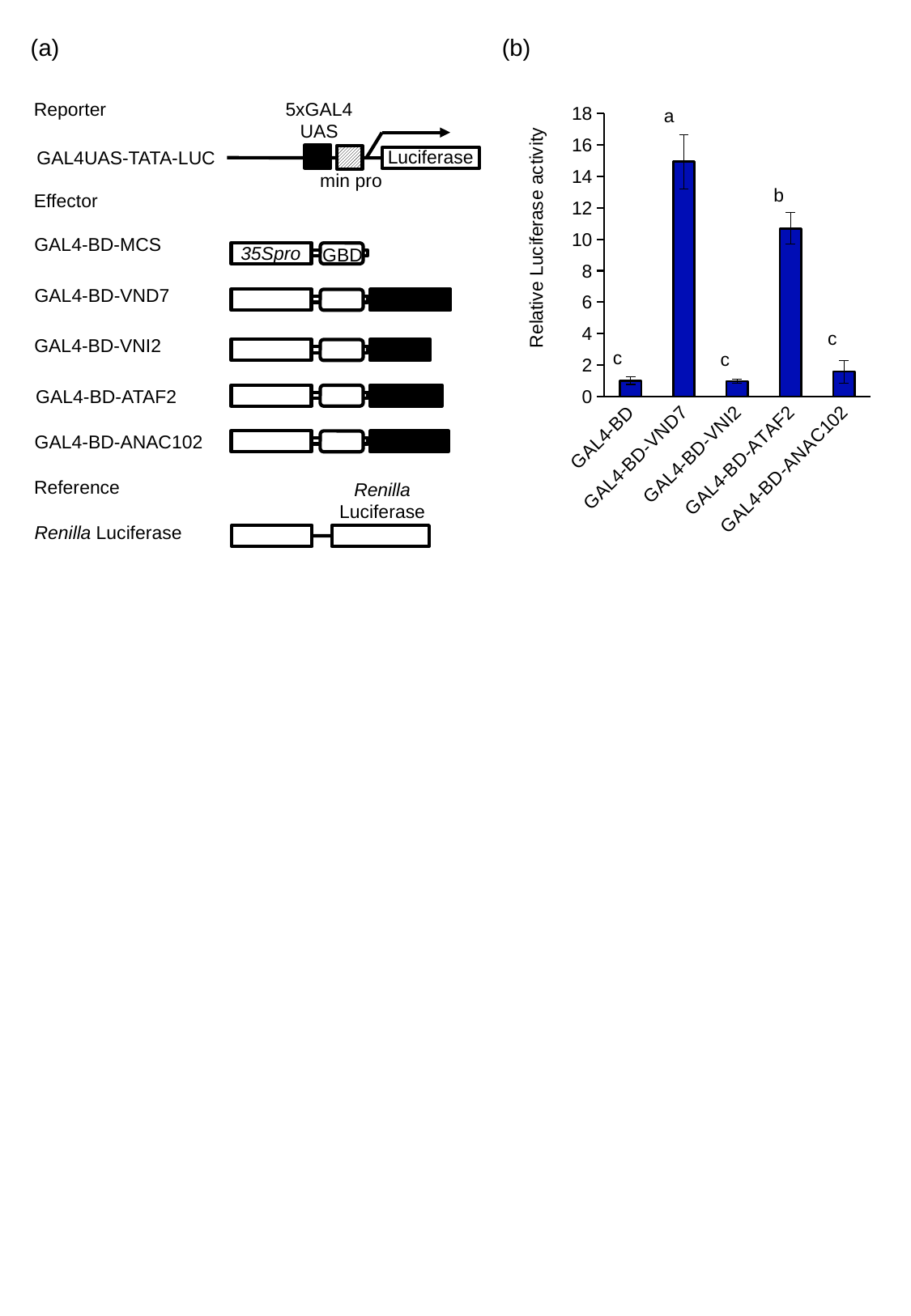

(a)
(b)
5xGAL4
UAS
Luciferase
min pro
Reporter
### Chart
| Category | |
|---|---|
| GAL4-BD | 1.0 |
| GAL4-BD-VND7 | 14.9429088877338 |
| GAL4-BD-VNI2 | 0.977017784259695 |
| GAL4-BD-ATAF2 | 10.6930431851128 |
| GAL4-BD-ANAC102 | 1.57894009304059 |a
b
c
c
c
GAL4UAS-TATA-LUC
Effector
GAL4-BD-MCS
35Spro
GBD
GAL4-BD-VND7
GAL4-BD-VNI2
GAL4-BD-ATAF2
GAL4-BD-ANAC102
Reference
Renilla
Luciferase
Renilla Luciferase
