# Supplementary material for: NAC domain transcription factors VNI2 and ATAF2 form protein complexes and regulate leaf senescence
Source: Plant Direct. 2023 Sep 18;7(9):e529. doi: 10.1002/pld3.529 (PMC10507225; doi:10.1002/pld3.529)
Supplement: Supplementary file 4 — Figure S3. The whole NAC domain of VNI2 is necessary for interaction with ATAF2. (a) Schematic diagram of full length and truncated VNI2 used for the yeast two‐hybrid assay. (b) Result of yeast two‐hybrid assay. Full length ATAF2 fused to GAL4‐BD, and full length or truncated VNI2 fused to GAL4‐AD were introduced into AH109 yeast cells. The transformed cells were grown on control (Trp−Leu−) and selective media (Trp−Leu−His− with .1 mM 3‐AT). Plasmids containing MCS fused to GAL‐BD or GAL4‐AD were used as negative controls, and pBD‐wt and pAD‐wt were used as positive controls. [file PLD3-7-e529-s005.pptx]

## Slide 1
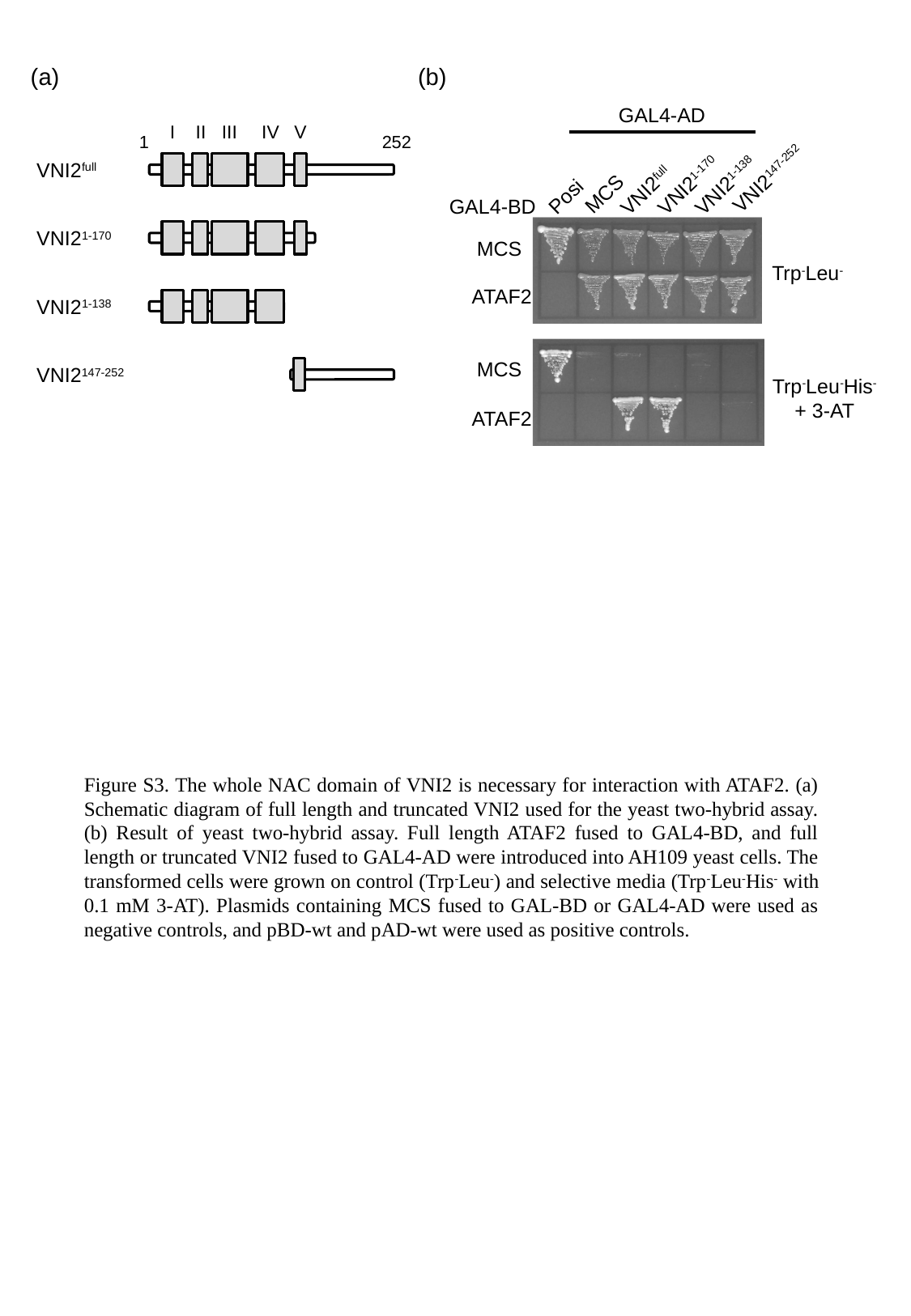

(a)
(b)
GAL4-AD
VNI2147-252
VNI21-170
VNI21-138
VNI2full
MCS
Posi
GAL4-BD
MCS
Trp-Leu-
ATAF2
MCS
Trp-Leu-His-
+ 3-AT
ATAF2
I
II
III
IV
V
1
252
VNI2full
VNI21-170
VNI21-138
VNI2147-252
Figure S3. The whole NAC domain of VNI2 is necessary for interaction with ATAF2. (a) Schematic diagram of full length and truncated VNI2 used for the yeast two-hybrid assay. (b) Result of yeast two-hybrid assay. Full length ATAF2 fused to GAL4-BD, and full length or truncated VNI2 fused to GAL4-AD were introduced into AH109 yeast cells. The transformed cells were grown on control (Trp-Leu-) and selective media (Trp-Leu-His- with 0.1 mM 3-AT). Plasmids containing MCS fused to GAL-BD or GAL4-AD were used as negative controls, and pBD-wt and pAD-wt were used as positive controls.
