# Supplementary material for: NAC domain transcription factors VNI2 and ATAF2 form protein complexes and regulate leaf senescence
Source: Plant Direct. 2023 Sep 18;7(9):e529. doi: 10.1002/pld3.529 (PMC10507225; doi:10.1002/pld3.529)
Supplement: Supplementary file 6 — Table S1. Oligonucleotides used in this study. [file PLD3-7-e529-s003.pdf]

Table S1. Oligonucleotides used in this study.

Primer	Primer Sequence（5’-3’）	Purpose
VNI2F	CACCATGGATAATGTCAAACTTGTTAAGAATGGT	For amplifying CDS sequence of *VNI2* gene
VNI2R	TCATCTGAAACTATTGCAACTACTGGTCTC	For amplifying CDS sequence of *VNI2* gene
ATAF2F	CACCATGAAGTCGGAGCTAAATTTACCA	For amplifying CDS sequence of *ATAF2* gene
ATAF2R	TTACCCCTGTGGAGCAAAACTCCAATTC	For amplifying CDS sequence of *ATAF2* gene
ORE1 promoter F	CACCTATCTCTATTAACGGTTACCATCAAAATTT	For amplifying promoter sequence of *ORE1* gene
ORE1 promoter R	GTAATCCATTTTATCCTAATAGGGTTTCTA	For amplifying promoter sequence of *ORE1* gene
ORS1 promoter F	CACCCCAGTCTTAGCTAGTTTGTTTTATTTAATT	For amplifying promoter sequence of *ORS1* gene
ORS1 promoter R	GTAATCCATTTCAGCGATTAGATATAATCA	For amplifying promoter sequence of *ORS1* gene
VNI2 promoter F	CACCGAAATCGATCATTTTTTTATTTTAATTTAG	For amplifying promoter sequence of *VNI2* gene
VNI2 promoter R	ATTATCCATGGTGGTTCCAAACAAAGAGAG	For amplifying promoter sequence of *VNI2* gene
Multiple cloning site (MCS) F	CACCTAGTGGATCCCCCGGGCTGCAGGAATTCGATATCAAGCTTATCGATACCGTCGACCTCGTGATG	For amplifying CDS sequence of MCS
Multiple cloning site (MCS) R	CATCACGAGGTCGACGGTATCGATAAGCTTGATATCGAATTCCTGCAGCCCGGGGGATCCACTAGGTG	For amplifying CDS sequence of MCS
ATAF2-RTF	TCTTCTACGCAGGGAAAGCTCC	Primer used for qRT-PCR
ATAF2-RTR	AGCCATTGTCGTGGTCCTCG	Primer used for qRT-PCR
ORE1-RTF	CTTACCATGGAAGGCTAAGATGGG	Primer used for qRT-PCR
ORE1-RTR	TCGGGTATTTCCGGTCTCTCAC	Primer used for qRT-PCR
ORS1-RTF	CCTTGGAAGGCTAAGCTTGG	Primer used for qRT-PCR
ORS1-RTR	ATCAGCGGAGGTAAACCAAC	Primer used for qRT-PCR
ANAC046-RTF	GGTTTCGGTTTCACCCTACTGATG	Primer used for qRT-PCR
ANAC046-RTR	ATGGCTCGTTCTTGTTGAGGTC	Primer used for qRT-PCR
VNI2-RTF	TTTGCAGAGCTGATCCTTGG	Primer used for qRT-PCR
VNI2-RTR	CGGTTCCCATTTGGGTATTT	Primer used for qRT-PCR
SAG13-RTF	AGGGAGCATCGTGCTCATATCC	Primer used for qRT-PCR
SAG13-RTR	CCAGCTGATTCATGGCTCCTTTG	Primer used for qRT-PCR
SAG29-RTF	TAAGCGCCGTTATGTGGTTCGC	Primer used for qRT-PCR
SAG29-RTR	ATCCCACCACGTTTGGAATCGC	Primer used for qRT-PCR
eIF4a-RTF	TGACCACACAGTCTCTGCAA	Primer used for qRT-PCR
eIF4a-RTR	ACCAGGGAGACTTGTTGGAC	Primer used for qRT-PCR
VNI2 - a	CACCATGGATAATGTCAAACTTGTTAAGAATGGT	Primer used for RT-PCR in S4
VNI2 - b	TCATCTGAAACTATTGCAACTACTGGTCTC	Primer used for RT-PCR in S4
ATAF2 - c	CACCATGAAGTCGGAGCTAAATTTACCA	Primer used for RT-PCR in S4
ATAF2 - d	TTACCCCTGTGGAGCAAAACTCCAATTC	Primer used for RT-PCR in S4
