# Supplementary material for: NAC domain transcription factors VNI2 and ATAF2 form protein complexes and regulate leaf senescence
Source: Plant Direct. 2023 Sep 18;7(9):e529. doi: 10.1002/pld3.529 (PMC10507225; doi:10.1002/pld3.529)
Supplement: Supplementary file 1 — Data S1. Peer Review. [file PLD3-7-e529-s004.docx]

**Editor**

Overall, I can readily see this is important work illuminating control of senescence. On the other hand, I would like to more clearly see in the abstract and discussion which assertions directly follow the evidence and which are new hypotheses that are not yet fully proven. This would address concerns from the reviewers about conclusions not being justified by results. Also please check the methods to ensure the work is fully reproducible by another lab.

-Thank you for carefully reading our manuscript and providing the positve and constructive comments. We checked the whole manuscript and revised it to clearly see which assertions directly follow the evidence and which are possibilities that are not yet fully proven.

In addition, we described the methods in more detail.

**REVIEWER COMMENTS:

Reviewer: 2

Reviewer Report for the Authors**
Overall this is an interesting work on the role of the VNI2-ATAF2 interaction in regulation of senescence. However, in my opinion, the conclusions are not yet justified by their results and additional data and experiments should be provided:

-Thank you for carefully reading our manuscript and giving us the valuable and critical comments.

1. The title does not really reflect their findings. They did not show that the complex is formed in senescent tissue which is stated in the title. They have shown that these two proteins can interact and that both are related to senescence regulation but not that an interaction takes place during senescence.

-Thank you for the valuable comments. We have revised the title to better reflect our findings to "NAC domain transcription factors VNI2 and ATAF2 form protein complexes and regulate leaf senescence."

2. Line 149: Why did the author use a library of root cells cDNA? Which other proteins that interact were identified? Did they also find VND7? Or more other NACs?. As they analyze the effect on leaf senescence, would it not make more sense to use a leaf library?

-We have shown that *VNI2* is expressed in various types of cells in roots (Yamaguchi et al. 2010). Therefore, we expected that some of interacting proteins with VNI2 should have been isolated from the cDNA library of root cells.

3. Lane 187/Figure 1c: In the text it is stated: "His-VNI2 protein was effectively retained on the resin incubated with MBP-ATAF2, while the resin immobilized with MBP hardly retained the His-VNI2 (Figure 1c). What is the difference between lane 2 and lane 4, both are incubated with MBP-ATAF2 and His-VNI2, but in lane 2 a weaker signal is observed than in lane 3 which is the negative control, while in lane 4 there is a much stronger signal even though lane 2 and 4 appear to be identical? This is not convincing!

-Thank you for carefully checked our manuscript. This was our fault. At lane 2 in Figure 1c, only His-VNI2 but not MBP-ATAF2 were included. We corrected the Figure 1c.

4. Figure 1d: For in vivo interaction a FRET or BiFC system should be used to show in vivo fluorescence also providing the localization of the interaction.

-We attempted to use the BiFC system to investigate in vivo interaction. However, we could not obtain fluorescence signals. VNI2 protein is known to be quite unstable. It is possible that this instability is the reason why we could not obtain the results. Therefore, we focused on the co-immunoprecipitation assay to show the in vivo interaction between VNI2 and ATAF2.

5. Figure 2a: They should indicate in the figure that they used the 35S promoter in all cases. Like it is now, it suggests that the promoter of VNI2, ATAF2 and the Renilla luciferase would be different.

-According to the reviewer's kind suggestion, we have revised Figure 2a.

6. Line 211/Figure 2b: In the text it is stated "These data indicated that VNI2 inhibits ATAF2 function by forming a protein complex." This is only clear for the ORE1 promoter, here VNI2 alone has no effect but together with ATAF2 it inhibits ATAF2 function. For the OSR1 and the VNI2 promoter, VNI2 alone has already a significant repressing function. Therefore, in these cases it is not clear, whether ATAF2 activation and VNI2 repression are two additive but independent effects or whether the proteins interact directly!!!!

-Thank you for carefully reading our manutscript. As the reviewer mentioned, VNI2 alone signifanctly reduced the reporter gene expression driven by the *VNI2* promoter. It is possible that the VNI2 interacts with and inhibits the endogenous transcription factors. We added the possiblity in the revised manuscript.

7. What about the NIT2 promoter which is negatively regulated by ATAF2. Which influence has VNI2 on the expression of a negatively regulated gene?
8. Line 226: Spontaneous mutants...? This makes no sense for me. Do they mean "simultaneous"?

-As the reviewer pointed, "Simultaneous" is correct. We corrected it in the revised manuscript.

9. Figure 2: the author should present different developmental stages of the plants so that it can be observed whether onset or progression of senescence is affected.

- For the transient expression assay, we prepared the protoplast cells from leaves of 3- to 4-week-old Arabidopsis plants without bolting. We did not choose the leaves obviously progressing senescence.

10. What about VNI2 overexpression in wild-type and in ataf2 mutant background?

Thank you for the valuable suggestion. We are planning to generate *VNI2* overexpression lines in both the wild-type and *ataf2* mutant backgrounds and plan to use them in future studies.

11. Line 229/ Figure 2b: According to the chlorophyll values, ataf2 mutant plants show delayed senescence, vni2 mutant plants accelerated senescence. The double mutant is intermediate, very similar to the wild-type. In the text it is stated that "These data indicated that early leaf senescence in vni2 mutants is dependent on the existence of ATAF2." However, this cannot be concluded from the chlorophyll data. This could also be the addition of independent effects!

-Thank you for the critical comment. We changed the description from "These data indicated that early leaf senescence in vni2 mutants is dependent on the existence of ATAF2." to "These data indicated that decrease of the chlorophyll content in *vni2* mutants is dependent on the existence of ATAF2."

12. Line 236/Figure 4: The author should comment on why in the vni2 mutant expression is higher compared to wild-type at 30 days but it is the opposite at 42 and 49 days. Moreover, the expression of the SAGs is also not as consistent as they describe it in the text. For SAG29, expression is lower in vni2 mutant and ataf2 mutant plants but higher in the double mutant at 42 days, but at 49 days expression is higher in vni2 mutants, still lower in ataf2 mutants and intermediate in the double mutant. So, the conclusion they have drawn in the text "These data suggested that VNI2 represses age-dependent leaf senescence through inhibition of senescence-associated genes regulated by ATAF2" is not really justified, as the interplay appears to be more complex.

-Thank you for the important comments. At first, we have shown that ATAF2 upregulates *ORE1*, *ORS1* and *ANAC046* by the transient expression analysis (Fig. 2) and expression analysis of ATAF2 overexpression plants (Nagahage et al. 2020). Then, Fig. 3 showed that expression of these genes were increased in *vni2* mutants than wild type plants. Taken together, we can say that "These data suggested that VNI2 represses age-dependent leaf senescence through inhibition of senescence-associated genes regulated by ATAF2". As the reviewers pointed, it is hard to interpretate the conclusion after description of the results of the *vni2ataf2* double mutants. Therefore, we moved the conclusion before the results of the *vni2ataf2* double mutants.

It is unclear how *ATAF2* expression is regulated. Therefore, it is difficult to explain why ATAF2 expression is higher in *vni2* mutants than that in wild type plants at 30 days but it is the opposite at 42 and 49 days. It is necessary to study the regulatory mechanisms of *ATAF2* expression in detail.

13. Line:259/Figure 5: "These data suggested that VNI2 delays dark-induced leaf senescence promoted by ATAF2". Again, this conclusion is not justified by the chlorophyll contents. The double mutant is intermediate and different from the wild-type, so that this could still be independent and just additive effects.

-According to the reviewer's opinion, we deleted the sentence "These data suggested that VNI2 delays dark-induced leaf senescence promoted by ATAF2".

14. Line:267/Figure 5: again, the expression of the NACs and the SAGs is also not as consistent as they describe it in the text and does not justify the conclusion "These data suggested that VNI2 represses the progression of dark-induced leaf senescence promoted by ATAF2.

-According to the reviewers comment, we changed the conclution to "These data suggested that VNI2 represses the dark-induced leaf senescence through inhibition of senescence-associated genes regulated by ATAF2". In addition, as in the case of the age-dependent leaf senescence, the conclusion was moved before the results of the *vni2ataf2* double mutants.

15. What about direct DNA-binding of VNI2? It has a NAC domain and a repressing EAR domain. In principle, it could also function as direct DNA binding protein?

-Thank you for the valuable comment. Honestly speaking, we have not concluded whether VNI2 directly binds to the promoter region of genes involved in leaf senescence or not. It is necessary to study that point in future.

16. Does the EAR domain of VNI2 recruit TOPLESS, like in hormone signaling complexes? Was TOPLESS found in the yeast two hybrid screen?

-We expected that some proteins which interacts with the EAR motif could be isolated through our screening. However, we could not isolate TOPLESS family as the interacting proteins.

17. Is xylem differentiation affected in the ataf2 mutant?

-Thank you for asking the interesting question. We have not observed xylem vessel formation in *ataf2* mutant. We will study whether ATAF2 affects xylem vessel differentiation.

18. Figure 7: VNI2 should be written in one line

-Thank you for carefully checked our manuscript. We corrected it.

**Reviewer: 4

Reviewer Report for the Authors**

Dear authors:
In this manuscript (ID: TPJ-00364-2023), you identified VNI2 and ATAF2 as leaf senescence related factors in Arabidopsis, and found ATAF2 could interact with VNI2 by using in vitro pull-down assay and in transient expression assay in N. benthamiana leaves and in Arabidopsis protoplasts. Mutant analyses data showed that VNI2 may inhibit ATAF2 activities, and caused repressing of leaf senescence progression in Arabidopsis.

It is a straight-forward study and I believe the manuscript is prepared well and main results support the initial design. The findings are meaningful and interesting for our understanding toward to the biological roles of VNI2 in leaf senescence. Thus, from a content point of view, I like the manuscript.

However, following comments are encouraged to consider or address when improving the manuscript:

-Thank you for carefully reading our manuscript and providing us the useful comments.

(1) For the molecular level data investigation of different Arabidopsis plants, you checked the up-regulated genes, ORE1, ORS1, ANAC046, SAG12, SAG13, SAG29, etc. Those down-regulated genes, such as CAB1, RBCS1A, should be included as well.

-Thank you for your valuable comment. In this study, we checked the genes, which we have shown that ATAF2 upregulates. It is known that ATAF1, closely related to ATAF2, downregulates genes involved in chlorophyll maintenance. It is expected that to study the genes associated with photosynthesis can be obtained new insights considering the biological meaning of the VNI2-ATAF2 interaction.

(2) It would be better to provide the data on overexpression observation of VNI2 and ATAF2 in both Columbia wild-type and mutant backgrounds.

-Thank you for the valuable suggestion. We are planning to generate *VNI2* overexpression lines in both the wild-type and *ataf2* mutant backgrounds and plan to use them in future studies.

(3) Please clarify the "spontaneous mutations" in Line 226-227.

-Thank you for carefully reading our manuscript. "Simultaneous mutations" is correct. We have corrected it in the revised manuscript.

**Reviewer: 1**

**Major Concerns:**
1. For Pull down assay, there are insufficient controls. And the VNI2 also can be pull down by MBP alone even the signal is much weaker than ATAF2-MBP.

-Thank you for carefully checked our manuscript and giving us the immportant comments. In the first version of our manuscript, there was a fault. At lane 2 in Figure 1c, only His-VNI2 but not MBP-ATAF2 or MBP were included. We corrected the Figure 1c. Even when only His-VNI2 was incubated, Amylose Resin slightly retained the His-VNI2 protein (lane 2). His-VNI2 signlas at lane 2 and 3 were obviously weaker than that at lane 4. Therefore, we thought that His-VNI2 signals at lane 4 was due to interaction between ATAF2 and VNI2 proteins.

2. For the Co-IP assay, the GFP negative control should add in because some proteins very easy to be co-immunoprecipitated by GFP-trapped beads.

-The members of our authors who are expert of co-immunoprecipitation assay did the experiments and repeatably obtained the result. We believe that the results of the Figure 1d indicated the in vivo interaction between ATAF2 and VNI2 proteins.

3. Does ATAF2 directly bind to promoter sequences of ORE1? Although the authors confirmed that ATAF2 up-regulated the expression of ORE1 and VNI2 in a LUC reporter system, however, evidence of ATAF2 directly bind to ORE1 promoter is critical to function of ATAF2. Therefor, EMSA and/or Y1H assay should be done to provide evidence of that.

-Thank you for the important coments. It has been reported that ATAF1, closely related to ATAF2, directly regulates *ORE1* expression. However, we have not concluded whether ATAF2 directly or indirectly regulates *ORE1* or *VNI2* expression. It is necessary to investigate that points in near future.

4. The natural leaf senescence of vni2 ataf2 is very strange. The ataf2 single mutant displayed same leaf senescence phenotype with Col-0, however, it can largely recover the early leaf senescence phenotype of vni2. How to explain?

-We previously showed that phenotypic changes were not obvious in the *ataf2* mutants in the developmental stage. It appears that ATAF2 has genetic redundancy with respect to the promotion of age-dependent leaf senescence. In this study, we showed that VNI2 inhibits ATAF2. Based on the conclusion, we could explain that the acceralated leaf senecence phenotype is observed in *vni2* mutants due to release of the ATAF2 activity inhibited by VNI2 while it is suppressed in *vni2ataf2* double mutants due to lack of ATAF2 function. It is also important that *vni2ataf2* double mutants partly but not completely recovered the acceralation of leaf senescence. This results could support the genetic redundancy of ATAF2.

5. ATAF2 is very close to ANAC102, so they probably function redundancy. Therefore, ataf2 anac102 double mutant should be obtained to confirm the function of them in the natural leaf senescence, and ataf1 anac102 vni2 triple mutant is necessary to determine the genetic relationship between VNI2 and ATAF2 during natural leaf senescence rather than ataf2 anac102 double mutant.

-Thank you for the atrractive idea. It is known that ATAF1 promotes leaf senescence as well as ATAF2. Therefore, we want to generate multiple mutants in combinations with *VNI2*, *ATAF1*, *ATAF2*, and *ANAC102* loci.

Reviewer: 2
Major Concerns: The conclusions drawn are not justified by the results of the authors. They might be correct but they are not yet proven.

-Thank you for the ciritical comment. We checked the whole manuscript and revised it to clearly see which assertions directly follow the evidence and which are possibilities that are not yet fully proven.

**Reviewer: 3**

**Major Concerns:**

In this research, Nagahage et al. identified two NAC transcription factors (TFs) ATAF2 and ANAC102 as physical interactors of another NAC TF VNI2, which suppresses xylem vessel formation and regulates leaf senescence. They provided biochemical, genetic, and molecular evidence to demonstrate that VNI2 is an inhibitor of ATAF2 and its regulation of downstream genes and leaf senescence.

-Thank you for carefully reading our manuscript.

1. It is most likely that ANAC102, as the closest homolog of ATAF2, is also involved in VNI2-ATAF2 module and function in xylem vessel formation and/or leaf senescence. The authors should analyze the three NACs as a whole to dissect their functions in a broader sense despite the observation that ANAC102 did not upregulate the LUC activity.

-Thank you for the valuable comment. To investigate how VNI2 regulates the interacting proteins, it is necessary to obtain the target genes which the interacting proteins up or downregulate. We are sorry but we could not find such kind of the target genes of ANAC102, and then we focused on the relationship between VNI2 and ATAF2. We would like to investigate the relashionship among VNI2, ATAF2, and ANAC102 in future.

2. The physical and/or genetic association of VND7 with VNI2/ATAF2/ANAC102 should be further investigated to make the story more extensive.

-Thank you for the attractive opinion. It is interesting for us to understand why VNI2 interacts with distinct NAC domain proteins and plays roles in various biologiacl events. To answer the questions, it is important to generate the multiple mutants among *VNI2*, ANAC-A subfamily, and VND family loci.

3. Since VNI2/VND7 are known to regulate xylem vessel formation. The involvement of ATAF2 and ANAC102 in this process should also be investigated.

- Thank you for your valuable opinions. We have not observed xylem vessel formation in *ataf2* mutant. It is also interesting for us to understand whether ATAF2 and ANAC102 affects xylem vessel differentiation.

4. To make the story more solid, expression changes of leaf senescence genes regulated by VNI2/ATAF2/VND7/ANAC102 should be examined in higher-order mutants.

-In this study, we have focused on the relationhip beween VNI2 and ATAF2 during leaf senescence. As described above, we are interested in the bioligical roles of VNI2. We agree with the reviewer's comment that we have to generate and study the higher-order mutants to answer the question. It is noteworthy that *vnd7* single mutants do not show any defects on xylem vessel formation. Therefore, it is necessary to generate multiple mutants toghether with other *VND* genes. At first, we will study whether ATAF2 and ANAC102 regulates xylem vessel formation, or whether VND7 affects leaf senesence. When we obtain the evidence of the cross-talk, we will generate the multiple mutant lines for further study.

5. Promoter element analysis of those leaf senescence genes should be performed to predict the binding sites of those NACs. Selected bindings should then be verified experimentally.

- We have found an ideal binding sequence of VND7. However, about 50 % identity to the ideal binding sequence is enough to bind to VND7, suggesting that it is sometime difficult to identify the binding sequence of NAC domain transcription factors (Tamura et al. 2019). We think that it is necessary to study the consensus binding sequences of ATAF2 and ANAC102 more deeply first.

**Reviewer: 3**

**Minor Concerns:**

6. The title is too simple to reflect the major findings in this manuscript.

-Thank you for the comment. We revised the title.

**Reviewer: 1**
**Methods explanation:**

Some addational methods need further support the conclusions.

-According to the reviewer's opinion, we described the methods in more detail.

**Reviewer: 3
Methods explanation:**

The methods are good enough for experiments performed in this research.

-Thank you for the positive comment.

**Reviewer: 3
Language explanation:**

Minor editing is necessary.

-Thank you for the comments. We carefully check our manuscript, and revised them.
